# Comparative Analysis of *FLC* Homologues in Brassicaceae Provides Insight into Their Role in the Evolution of Oilseed Rape

**DOI:** 10.1371/journal.pone.0045751

**Published:** 2012-09-27

**Authors:** Xiaoxiao Zou, Ida Suppanz, Harsh Raman, Jinna Hou, Jing Wang, Yan Long, Christian Jung, Jinling Meng

**Affiliations:** 1 National Key Laboratory of Crop Genetic Improvement, Huazhong Agricultural University, Wuhan, China; 2 Plant Breeding Institute, Christian-Albrechts-University of Kiel, Kiel, Germany; 3 EH Graham Centre for Agricultural Innovation (an alliance between the Charles Sturt University and New South Wales Department of Primary Industries), Wagga Wagga Agricultural Institute, Wagga Wagga, New South Wales, Australia; National Taiwan University, Taiwan

## Abstract

We identified nine *FLOWERING LOCUS C* homologues (*BnFLC*) in *Brassica napus* and found that the coding sequences of all *BnFLCs* were relatively conserved but the intronic and promoter regions were more divergent. The *BnFLC* homologues were mapped to six of 19 chromosomes. All of the *BnFLC* homologues were located in the collinear region of *FLC* in the *Arabidopsis* genome except *BnFLC.A3b* and *BnFLC.C3b,* which were mapped to noncollinear regions of chromosome A3 and C3, respectively. Four of the homologues were associated significantly with quantitative trait loci for flowering time in two mapping populations. The *BnFLC* homologues showed distinct expression patterns in vegetative and reproductive organs, and at different developmental stages. *BnFLC.A3b* was differentially expressed between the winter-type and semi-winter-type cultivars. Microsynteny analysis indicated that *BnFLC.A3b* might have been translocated to the present segment in a cluster with other flowering-time regulators, such as a homologue of *FRIGIDA* in *Arabidopsis.* This cluster of flowering-time genes might have conferred a selective advantage to *Brassica* species in terms of increased adaptability to diverse environments during their evolution and domestication process.

## Introduction

*FLOWERING LOCUS C* (*FLC*) is a key regulator of flowering time in *Arabidopsis thaliana. FLC* and *FRIGIDA* (*FRI*) are important determinants of variation in the requirement for vernalization (i.e., the acceleration of flowering time in response to an extended period of low temperature) that is observed among natural ecotypes of *Arabidopsis* [1]–[3]. *FLC* encodes a MADS-domain transcriptional regulator that delays flowering by repressing the expression of floral integrators such as *FT*, *SOC1*, and *FD* in *Arabidopsi*s [4]–[6]. *FRI* upregulates *FLC* expression through at least two distinct pathways, which involve increased methylation of H3K4 and a co-transcriptional process to regulate efficient splicing of the *FLC* locus [7]–[10]. *FLC* is negatively regulated by autonomous pathway regulators, which are thought to act through chromatin remodeling or RNA processing, and is also repressed by vernalization pathway regulators during and after cold exposure [11]. Recent studies have revealed that a number of chromatin modifiers including long noncoding RNAs and polycomb components are involved in vernalization-mediated epigenetic silencing of *FLC* [12]–[17]. A 272-bp region in the promoter of *AtFLC* (*FLC* in *Arabidopsis thaliana*) is required for the cold-induced repression of the gene, and a 75-bp segment in this region is essential for expression of *AtFLC* in the absence of vernalization [18].

Cultivated *Brassica* species comprise many important vegetable, oil, food, and feed crops, which have evolved as a result of single or multiple polyploidization events after divergence from *Arabidopsis* lineages. Monogenomic diploid *Brassica* species, such as *B. rapa* (AA), *B. nigra* (BB), and *B. oleracea* (CC), underwent whole-genome triplication and 24 conserved collinear blocks (from A to X) of the ancestral karyotype (*n* = 8) have been identified in *Arabidopsis* and *Brassica* lineages [19]. Allotetraploid rapeseed (*Brassica napus* L., 2*n* = 4*x* = 38, AACC), a major oilseed crop that originated from natural hybridization between *B. rapa* and *B. oleracea*, shows a high degree of collinearity to its diploid progenitors [20]–[22]. However, as a result of polyploidization, a number of genes in *B. napus* have undergone gene duplication and subsequent nonfunctionalization or functional divergence, such as neofunctionalization or alternative gene expression [23], [24].

The *A. thaliana* genome contains one *FLC* gene. However, the diploid members of *Brassica* contain multiple copies of *FLC* and several such homologues have been shown to be associated with flowering time variation, such as *BrFLC.A10* (*BrFLC1*) and *BrFLC.A2* (*BrFLC2*) in *B. rapa* [25], [26] and *BoFLC.C2* (*BoFLC2*) in *B. oleracea* [27]. In *B. napus,* cDNA sequences of five *FLC* homologues (*BnFLC1* to *BnFLC5*) have been isolated and their ectopic expression delays flowering in *A. thaliana* [28]. However, it is unknown whether additional *FLC* homologues are present in the *B. napus* genome. Molecular mapping studies have revealed a number of quantitative trait loci (QTL) that control flowering time, and might involve *FLC* homologues, in different biparental populations of *B. napus* [29], [30]. Recently, allelic variation of *BnFRI.A3* (*FRIGIDA,* designated *BnaA.FRI.a*) was shown to be associated with flowering-time variation in a worldwide collection of *B. napus* accessions [31]. These findings suggest that the homologues of *FLC* and *FRI* play key roles in the regulation of flowering time in *B. napus*.

Various computational methods have been used to investigate potential gene regulatory elements from diverse organisms [32]. The comparison of promoter and intragenic regions of *BoFLC* genes in *B. oleracea* with *AtFLC* revealed extensive differences in structure and organization, but showed high levels of conservation within those segments essential for regulation of *FLC* expression [33]. This suggests that similar regulatory *cis*-acting elements were maintained during evolution even in duplicated genes that might have undergone functional divergence. In the present study, we characterized nine members of the *BnFLC* gene family and investigated their roles in the regulation of flowering time in *B. napus* via gene expression analysis. We also found that the *BnFLC.A3b* gene is tightly linked to a cold-responsive gene *BnCBF.A3*, and loosely linked to another flowering repressor gene, *BnFRI.A3.* Linkage of functionally related genes might have conferred a selective advantage during the evolution and domestication of *Brassica* species.

## Results

### Conservation and Divergence of *FLC* Homologues in Different *Brassica* Genomes

**Figure 1:**
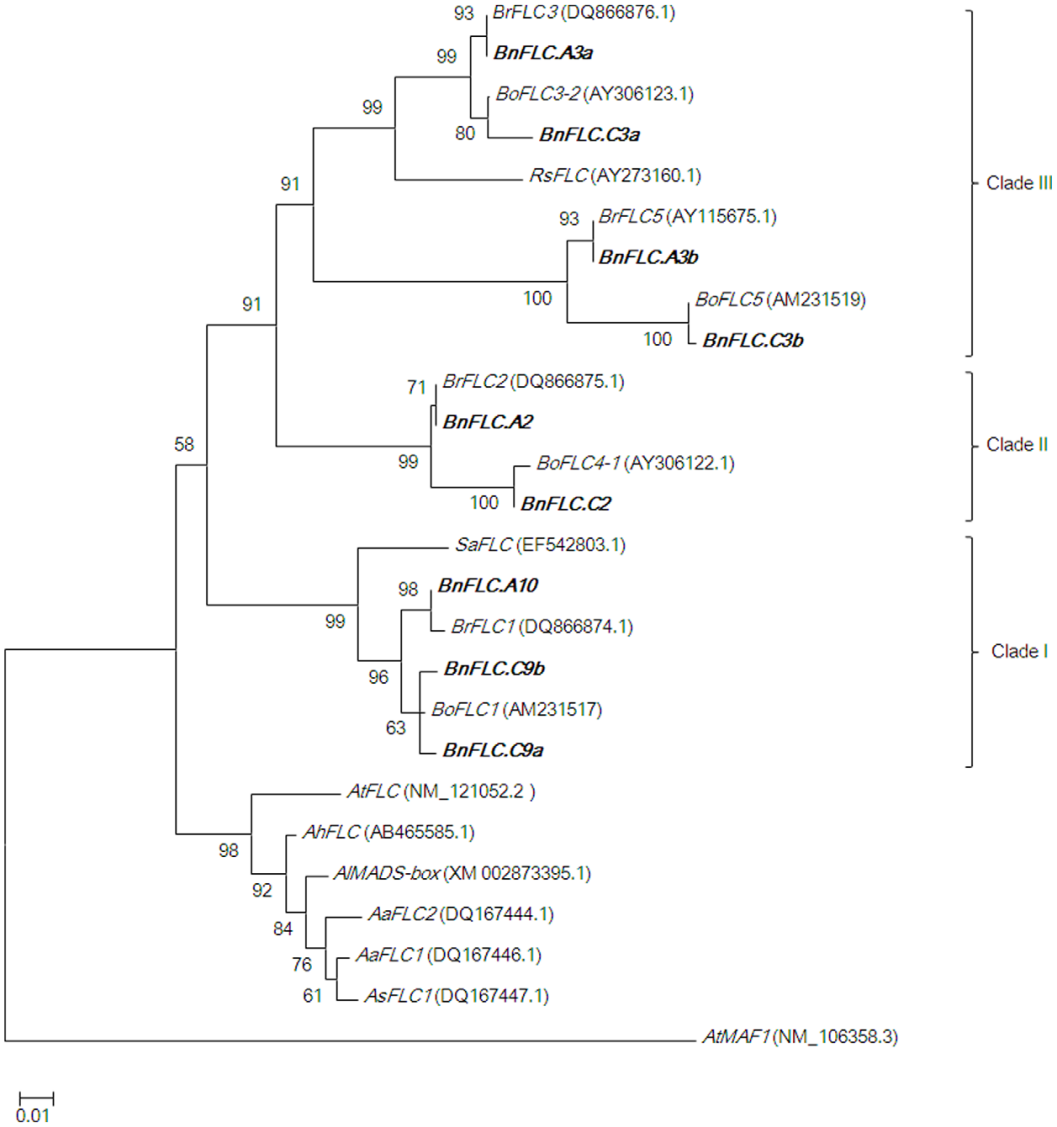
Phylogenetic tree of *FLC* homologues from *Brassica*, *Arabidopsis*, *Raphanus*, and *Sinapis* species. *BnFLC* homologues are highlighted in bold, and *AtMAF1* (*MADS AFFECTING FLOWERING 1* of *A. thaliana*, an *AtFLC*-like gene) was used as the outgroup. *Br*, *Brassica rapa*; *Bo*, *B. oleracea*; *Rs*, *Raphanus sativus* (radish); *Sa*, *Sinapis alba* (white mustard); *At*, *Arabidopsis thaliana*; *Al*, *A. lyrata*; *Ah*, *A. halleri*; *Aa*, *A. arenosa; As, A. suecica*. GenBank accession numbers are given in parentheses. Bootstrap support values are shown beside the branches.

**Figure 2:**
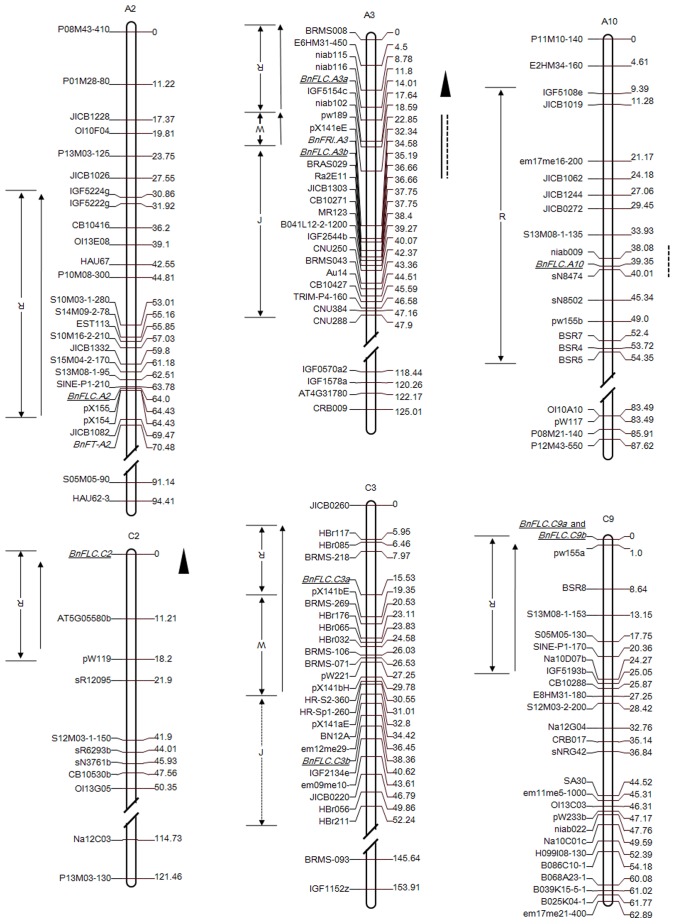
Location of *BnFLC* homologues and flowering-time QTL on the partial linkage map for TN-DH population. Genome blocks related to *BnFLC* homologues (underlined) were defined by comparative mapping of *B. napus* and *A. thaliana* and using the locus boundaries of the blocks reported by Schranz *et al.* [19]. Arrows at left indicate an opposite orientation of the blocks, and a dashed line represents undefined boundaries. Solid and dashed vertical bars on the right represent the position of flowering-time QTL colocalized with *BnFLC* homologues under winter- and spring-cropping environments, respectively. Triangles indicate the approximate position of flowering-time QTL associated with *BnFLC* genes, which were detected from the Skipton/Ag-Spectrum population.

**Figure 3:**
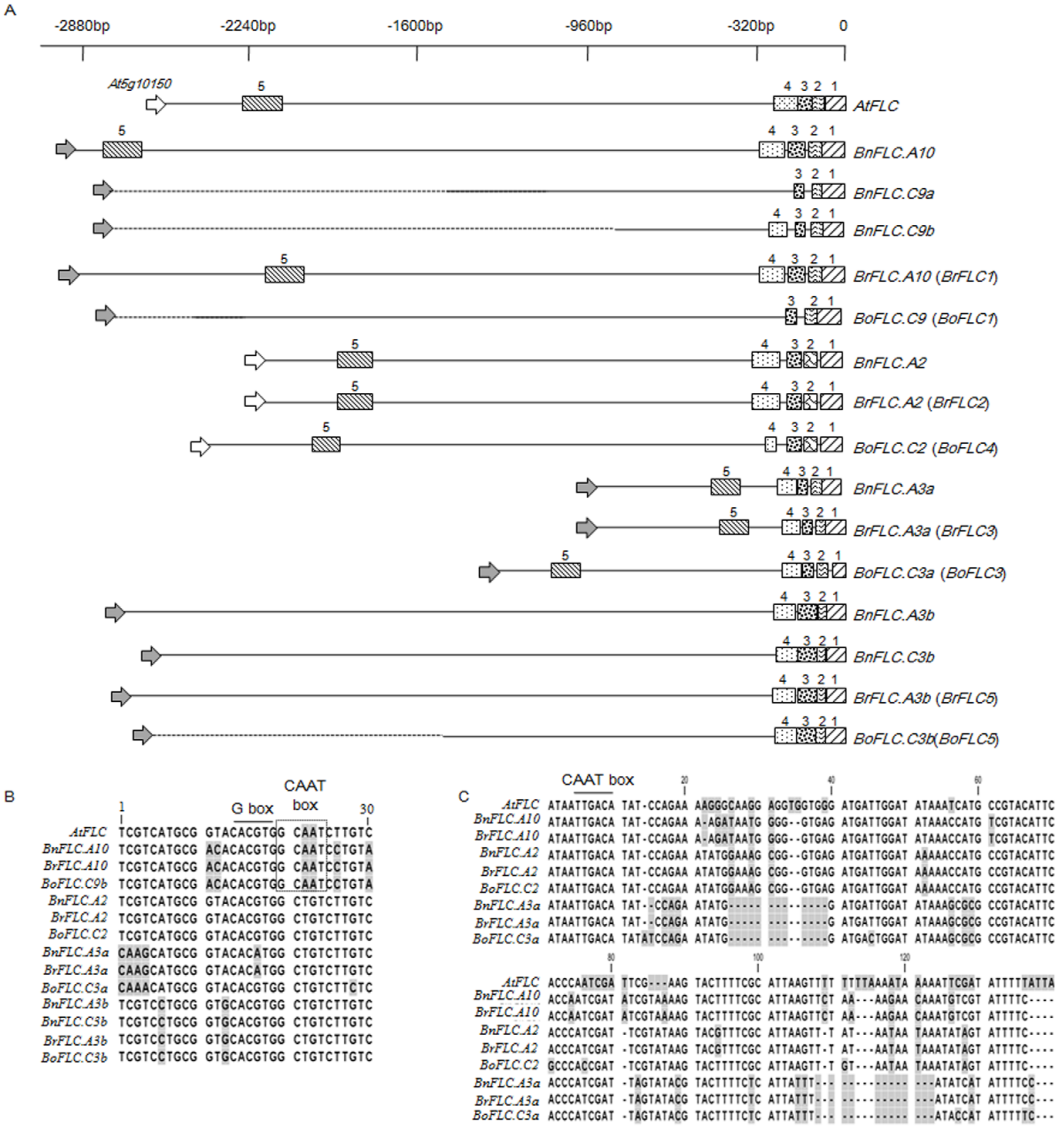
Analysis of the upstream regions of *Brassica FLC* homologues and *AtFLC.* (A) Comparison of the potential conserved *cis*-blocks upstream of *AtFLC* and *Brassica FLC* (0 to −2880 bp) homologues. Different conserved segments (more than 75% sequence identity, and referred to as *cis*-blocks in the text) are shown as boxes with different shading and are numbered for comparison. Arrows at the 5′ end indicate the approximate position of the neighbour gene (*At5g10150* and its homologues; unfilled) or the gene fragment (grey) upstream of *FLC.* Dashed lines represent upstream sequences that were not determined. (B) Alignment of 30 bp sequences that contain putative *cis*-regulatory elements (G-box and CAAT-box) in *cis*-block 4 among *FLC* homologues. (C) Alignment for the sequences in *cis*-block 5 among *FLC* homologues. The position of the putative CAAT-box (in the minus strand) is shown.

We cloned nine *FLC* homologues, four from the A genome and five from the C genome of *B. napus*, either from bacterial artificial chromosomes (BACs) or from PCR amplicons generated from genomic DNA (gDNA). We then compared their sequences to the homologues in their diploid progenitors *B. rapa* and *B. oleracea* (Table 1). Neighbour-joining cluster analysis using coding sequence data for *FLC* homologues among 10 species of Brassicaceae revealed that the *BnFLC* homologues were grouped into three distinct clades, which reflects the whole-genome triplication events that occurred during the evolution of the *Brassica* genome (Figure 1). Seven of the *BnFLC* homologues were located in the collinear ‘R’ block region (in which *FLC* is located in *Arabidopsis*) and included a tandem repeat on chromosome C9, whereas two homologues (*BnFLC.A3b* and *BnFLC.C3b*) were mapped to the noncollinear ‘J’ block (Table 1; Figure 2). Consistent with their homologues in other Brassicaceae, all *BnFLC* genes consisted of seven exons, which were interrupted by six introns and showed extremely high levels of similarity (95–100%) to the homologous sequences in the diploid progenitors *B. rapa* and *B. oleracea*. However, the promoter sequences of the *BnFLC* homologues were more divergent among each other and from *AtFLC* (Figure 3A). The 272-bp *cis*-regulatory region of the *AtFLC* promoter, which was reported to be conserved in *B. oleracea* [33], could be dissected further into four small blocks (referred to as *cis*-blocks) by sequence comparison in the present study. The first three *cis*-blocks were the most conserved among all *Brassica FLC* promoters, and the fourth *cis*-block, which corresponded to the crucial 75-bp segment for expression of *AtFLC* in nonvernalized plants [18], was absent from *BnFLC.C9a* and *BoFLC.C9.* A 30-bp segment that contained a G-box and a CAAT-box in *cis*-block 4 was highly conserved in some of the *BnFLC* homologues, as well as *AtFLC* (Figure 3B).

**Table 1:** *Brassica napus FLC* homologues, their map positions, and sequence identities compared with their orthologues in *B. rapa* or *B. oleracea.*

*BnFLC* homologue	Source^a^	Isolated region^b^	Orthologue in *B. rapa*or *B. oleracea*	Identity (%)^c^		
				Promoter region (approximately −1.5 kb)	Coding region	Intronic region
*BnFLC.A2*	JBnB035G21^*^	Promoter-3′ UTR	*BrFLC2* (A2, R)^d^	99	99 (0)^e^	99
*BnFLC.A3a*	JBnB50A15^*^	Promoter-3′ UTR^f^	*BrFLC3* (A3, R)	98	100 (0)	99
*BnFLC.A3b*	JBnB142O14^*^	Promoter-3′ UTR	*BrFLC5* (A3, J)	99	100 (0)	99
*BnFLC.A10*	JBnB75D10^*^	Promoter-3′ UTR	*BrFLC1* (A10, R)	100	99 (0)	99
*BnFLC.C2*	gDNA (T)	Exon 2-intron 6	*BoFLC4* (C2, R)	–	100 (0)	97
*BnFLC.C3a*	gDNA (N)	Exon 1-intron 1	*BoFLC3* (C3, R)	–	–	98
*BnFLC.C3b*	JBnB090B17^*^	Promoter-3′ UTR	*BoFLC5* (C3, J)	100	99 (0)	99
*BnFLC.C9a*	JBnB003G07^*^	Promoter-intron 6	*BoFLC1* (C9, R)	95	99 (0)	98
*BnFLC.C9b*	JBnB003G07^*^	Exon 1-intron 6	*BoFLC1* (C9, R)	–	99 (2)	98

a The sequences isolated from BACs are indicated by an asterisk, and those isolated from PCR-amplified genomic DNA are designated ‘gDNA’ with the parent indicated in parentheses (T, *B. napus* cv. Tapidor; N, *B. napus* cv. Ningyou7).

b The obtained sequences were compared with that of *AtFLC* to define the regions of each *BnFLC* gene that were isolated.

c To calculate sequence identities, indels (insertions or deletions) were excluded and for partial sequences of *BnFLC.C2* and *BnFLC.C3a,* the available region was used.

d The letter and numeral in parentheses represent the linkage group followed by the ancestral block in which the *FLC* homologues are located.

e The number of nonsynonymous nucleotide substitutions is shown in parentheses.

f There was a fragment with higher-order structure in intron 1 of *BnFLC.A3a* for which we failed to obtain the sequence.

Interestingly, another region (*cis*-block 5) was identified in the promoter of nine of the 16 *Brassica FLC* homologues. The region was located 2.3 kb upstream from the transcription start site of *AtFLC* and was well conserved between most *Brassica FLC* genes and *AtFLC.* Its size ranged from 115 to 142 bp (Figure 3C). A CAAT-box was found within this region, which is an indication of transcription factor binding activity.

### Transcriptional Divergence of *BnFLC* Homologues

*BnFLC* cDNA was amplified with gene-specific primers (Table S1) and subsequently sequenced. In total, eight different sequences were identified by multiple sequence comparison. Six of the sequences were isolated from both Tapidor and Ningyou7. The transcripts of *BnFLC.C2* and *BnFLC.C3a* were only isolated from a spring-type cultivar, Westar (Table S2). No *BnFLC.C3b* transcript was identified in Tapidor, Ningyou7 or Westar. *BnFLC.C3b* might be a pseudogene because it contains stop codons created by inserted nucleotides in exon 2 and exon 7 (Figure S1A).

**Figure 4:**
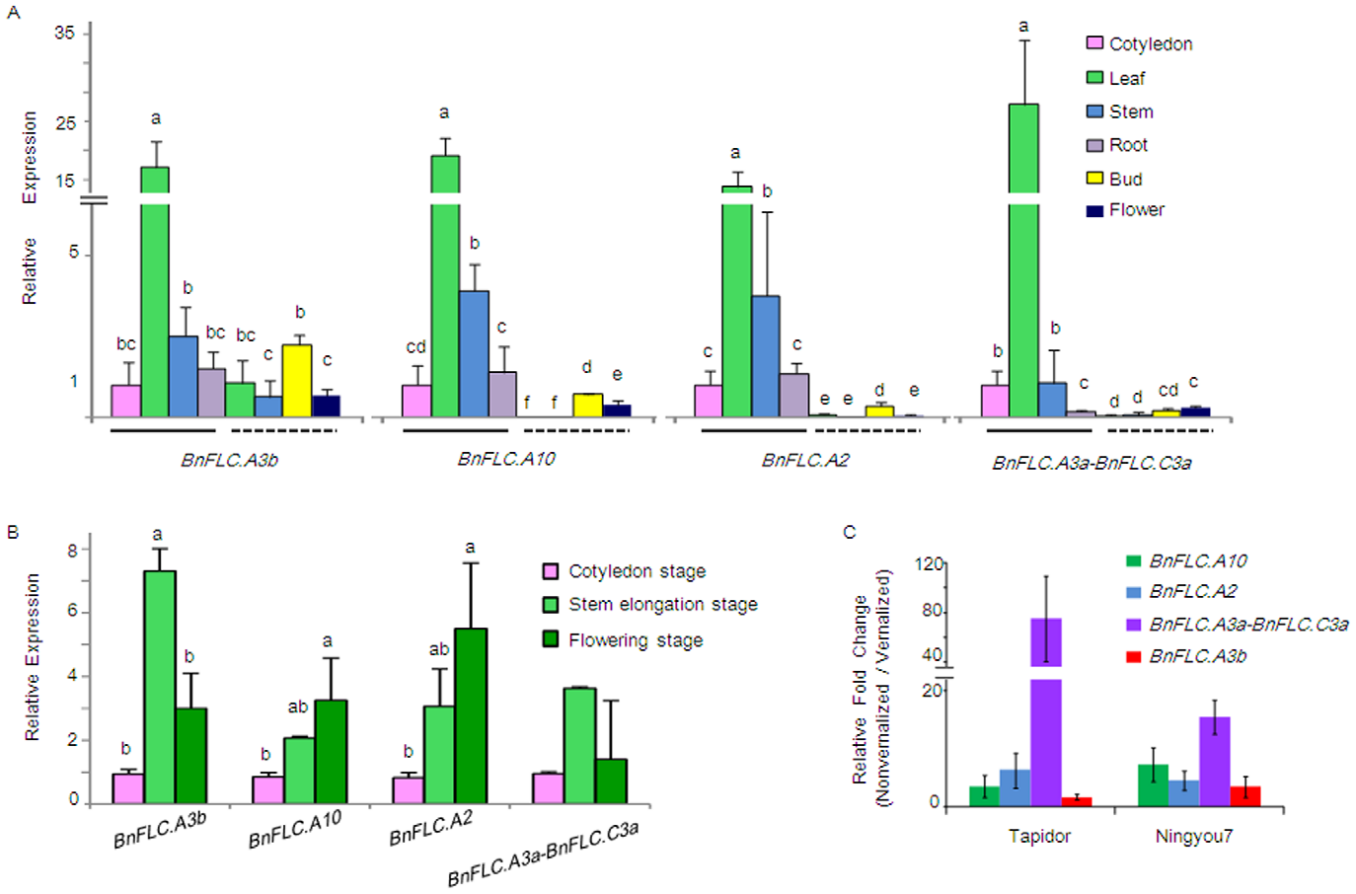
Quantitative real-time PCR analysis of expression patterns for four *BnFLC* homologues. Different letters above a bar indicate a significant difference (*P*<0.05). Expression levels at the cotyledon stage were considered to be the control. Relative expression values of each *BnFLC* homologue were normalized with the reference gene *β-Actin.* (A) Comparison of the relative expression levels of *BnFLC* homologues in different tissues of the winter cultivar Tapidor. Samples underlined with a solid or dashed line were collected from nonvernalized and vernalized plants, respectively. (B) Relative expression levels of *BnFLCs* in leaves and cotyledons at different developmental stages in the semi-winter cultivar Ningyou7. No cold treatment was applied throughout all of the developmental stages. (C) Vernalization responsiveness of *BnFLC* homologues in Tapidor and Ningyou7. The relative fold change between four-week-old leaves (without vernalization) and seven-week-old leaves (four-week-old plants followed by three weeks of cold treatment) was measured.

We selected four *BnFLC* homologues, *BnFLC.A2*, *BnFLC.A3b*, *BnFLC.A10*, and *BnFLC.A3a*-*BnFLC.C3a* for further analyzes. We would not design gene-specific primers for the other copies which were suitable for quantitative real-time PCR (qRT-PCR) due to the high sequence similarity among multiple copies. First, the relative expression levels of these *BnFLC* homologues were analyzed in different organs of the winter cultivar Tapidor at three developmental stages. Distinct expression patterns were distinguished. Without vernalization, the *BnFLC* homologues were highly transcribed in leaves, moderately transcribed in stems, and weakly transcribed in cotyledons and roots (Figure 4A). Different copies showed distinct relative expression levels in different organs. The expression of *BnFLC.A3a-BnFLC.C3a* was extremely high in leaves, and very low in roots, whereas *BnFLC.A10* and *BnFLC.A2* were highly expressed in leaves and stems. After vernalization, *BnFLC* transcriptional activity dropped sharply to very low levels in vegetative organs but was still detectable in reproductive organs (flower buds and fully-opened flowers).

Next, we analyzed the temporal expression profile of *BnFLC* homologues in cotyledons and leaves at different developmental stages [34] in the cultivar Ningyou7 (a semi-winter type), because it flowers without vernalization under long-day conditions. Compared with the cotyledon stage, *BnFLC* transcript abundance increased markedly at the stem elongation stage (Figure 4B). It was notable that, at the flowering stage, the *BnFLC* homologues were still highly expressed in nonvernalized Ningyou7 plants, which was in contrast to their repression in leaves at the flowering stage of vernalized Tapidor plants. The transcriptional activities of *BnFLC.A2* and *BnFLC.A10* were increased slightly at the flowering stage, whereas *BnFLC.A3b* was downregulated significantly compared with the stem elongation stage. Moreover, we found differences in the transcriptional activities of the *BnFLC* homologues after cold treatment for three weeks; all *BnFLC* genes were transcriptionally downregulated. However, *BnFLC.A3b* expression decreased at the slowest rate under cold treatment for 3 weeks in both Tapidor and Ningyou7, which suggested that *BnFLC.A3b* responds differently to cold than the other *BnFLC* homologues (Figure 4C).

### Sequence Differences between *BnFLC.A3b* Alleles and Differential Splicing

**Figure 5:**
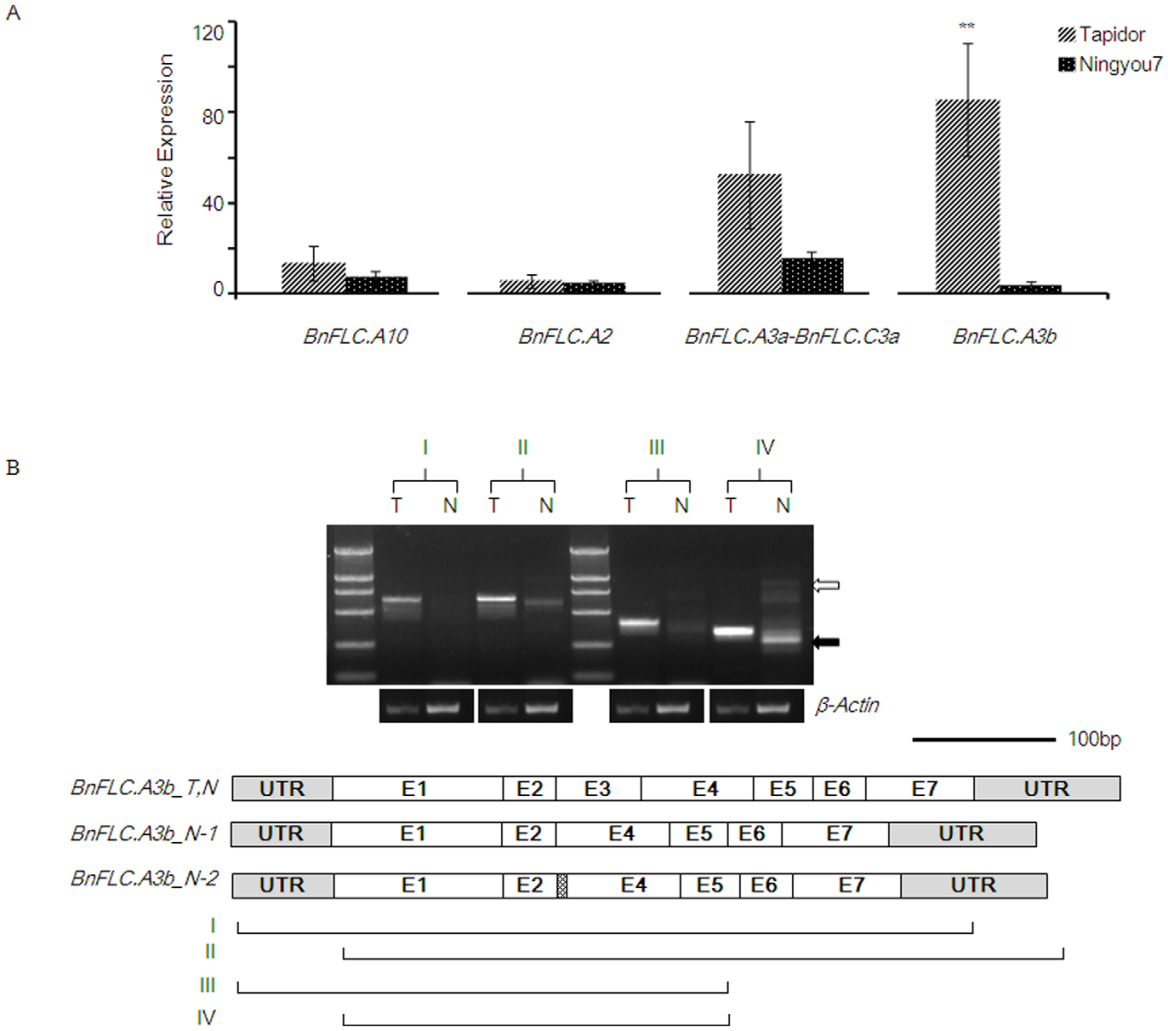
Differential expression of *BnFLCs* in four-week-old nonvernalized leaves from *B. napus* cultivars Tapidor and Ningyou7. (A) Relative expression levels for four *BnFLC* homologues. *β-Actin* was used as a reference gene. Error bars represent standard errors among the three biological replicates; **represents significantly different (*p*<0.05). (B) PCR products of *BnFLC.A3b* amplified from total RNA of Tapidor (T) and Ningyou7 (N). The major band amplified from Tapidor comprises correctly spliced *BnFLC.A3b* transcripts, whereas incompletely (unfilled arrow) and incorrectly spliced *BnFLC.A3b* transcripts (usually lack exon 3; filled arrow) were amplified in Ningyou7. The schematic diagram of a *BnFLC.A3b* transcript shows exons (E1 to E7) and untranslated regions (UTR). *BnFLC.A3b_T, N* represents correctly spliced transcript in Tapidor and Ningyou7, *BnFLC.A3b_N-1* and *BnFLC.A3b_N-2* represent the two kinds of alternative spliced transcripts in Ningyou7 which lacked partial or complete exon 3, respectively. Shadow box in *BnFLC.A3b_N-1* indicates the last four nucleotides of exon 3 which were retained. I, II, III and IV, which represent four amplifications from different transcriptional regions of correctly spliced *BnFLC.A3b_T, N* transcript, are shown below the schematic diagram.

Four QTL that control flowering time have been identified in the vicinity of *BnFLC.A3b* and *BnFLC.A10* (TN-DH population), and of *BnFLC.A3a* and *BnFLC.C2* (Skipton/Ag-Spectrum DH population), respectively (Figure 2). In the present study, we compared the expression levels of *BnFLC* alleles in four-week-old leaves from Tapidor and Ningyou7 plants to test whether the alleles were expressed differentially between the two parents of the TN-DH population (Figure 5A). However, only *BnFLC.A3b* allele showed significant expression differences. Twenty-five-fold higher expression of *BnFLC.A3b* was observed in the winter-type rapeseed parent compared with that in Ningyou7. It was notable that both *BnFLC.A3b* and *BnFRI.A3* were located within the confidence intervals of flowering-time QTL under both conditions, namely with and without cold treatment. To investigate the basis of the differential expression of the *BnFLC.A3b* alleles, we compared genomic *BnFLC.A3b* sequences from Tapidor and Ningyou7. Although a few indels and nonsynonymous single nucleotide polymorphisms were detected in intronic and regulatory regions (Figure S1B), these variations did not seem to contribute to the differences in expression of the two *BnFLC.A3b* alleles.

Next, we analyzed the DNA methylation status of *BnFLC.A3b* from −393 bp, in the promoter region, to +202 bp, at the 5′ end of the first intron, which covers the two CG islands of *BnFLC.A3b*. We used leaves from nonvernalized plants of Tapidor and Ningyou7. No obvious differences in DNA methylation were observed between the *BnFLC.A3b* alleles, and only a few cytosine sites at the 5′ end of the analyzed sequence were methylated (Table S3).

Finally, the *BnFLC.A3b* transcripts were analyzed with regard to splice site variations. Differential splicing was observed in nonvernalized leaves between Tapidor and Ningyou7 at the seedling stage. The transcripts from Tapidor were usually spliced correctly, but a greater number of incomplete or incorrectly spliced transcripts were identified in Ningyou7 than in Tapidor by semi-quantitative RT-PCR (Figure 5B) and sequencing of the PCR products. Further sequence analysis revealed that the mature *BnFLC.A3b* transcripts in Ningyou7 often lacked the complete or partial exon 3 (just retained the last four nucleotides). The latter transcript contained a premature stop codon, which suggests that the polypeptide might be non-functional (Figure S2). In conclusion, the amount of functional transcripts differed between both rapeseed types because of inefficient pre-mRNA processing in the semi-winter rapeseed.

### *BnFLC.A3b* Resides within a Cluster of Cold Responsive Genes

We analyzed the sequences that bordered the *BnFLC.A3b* and *BnFRI.A3* genes with an aim to investigate the genomic structure between these two flowering-time genes. In contrast to their close linkage in *B. napus*, these genes are unlinked in *A. thaliana* and *A. lyrata* (Figure S3). *BnFLC.A3b* and *BnFRI.A3* were located in close proximity to each other on chromosome A3 (Figure 2). This genomic region is collinear to neither the ‘R’ block, which contains *AtFLC* (At5_3.5 Mb), nor the ‘O’ block, which contains *AtFRI* (At4_0.2 Mb).

**Figure 6:**
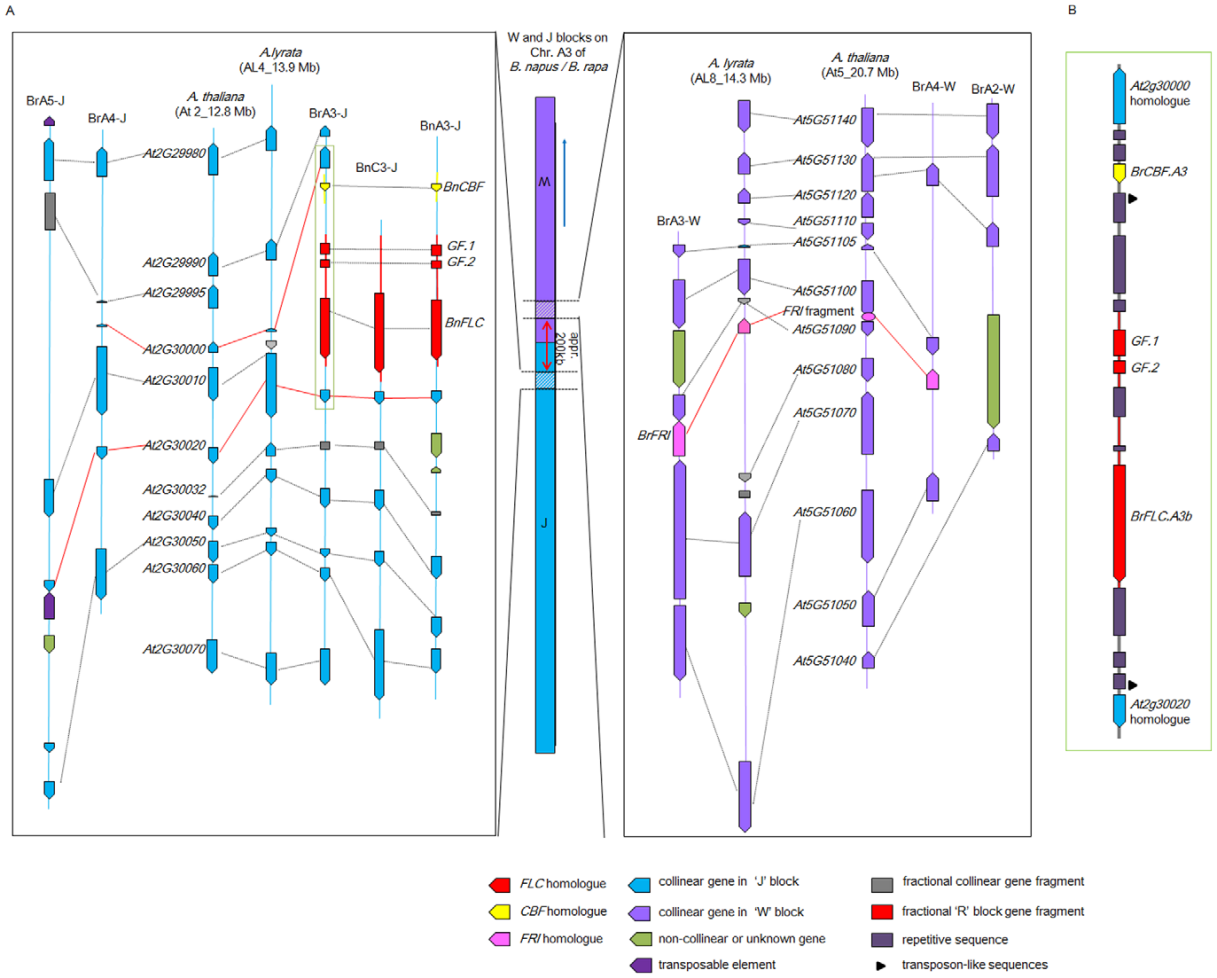
Microsynteny analysis of homologous regions related to *BnFLC.A3b*, *BnFRI.A3*, and *BnCBF.A3*. Collinear gene models are connected by dotted lines. (A) The colored bar in the center represents the portion of chromosome 3 of *B. napus* that contains the three genes. The blue arrow indicates the opposite orientation of the ‘W’ block. The left-hand box represents a region of the ‘J’ block (BnA3-J) in which *BnFLC.A3b* and *BnCBF.A3* are located, and alignment of conserved gene models of BnC3-J of *B. napus,* the triplicated regions (BrA3-J, BrA4-J and BrA5-J) of *B. rapa*, and the homologous regions in *A. thaliana* and *A. lyrata*. Red lines connect the collinear gene models in the ‘J’ block next to *FLC* and *CBF* homologues. The right-hand box represents a region of the ‘W’ block in which *BnFRI.A3* is located, and alignment of conserved gene models in the triplicated regions (BrA2-W, BrA3-W and BrA4-W) of *B. rapa,* and the homologous regions in *A. thaliana* and *A. lyrata*. Red lines connect the *FRI* homologues and the *FRI* homologous fragments. (B) Detailed structure of a region in BrA3-J (indicated by a green box in the left-hand box of (a) from the BAC clone KBrH038M21; [35]). Repeat sequences were identified between the homologues of *At2g30000* and *At2g30020*. *GF-1* and *GF-2* represent two gene fragments homologous to the ‘R’ block genes, *At5G10200* and *At5G10150*, respectively.

First, we compared the *BnFLC.A3b* and *BnFLC.C3b* loci with the corresponding triplicated segments from the ‘J’ block in *B. rapa*, and with the orthologous genomic regions in *A. thaliana* and *A. lyrata* (Figure 6A). Significant collinearity at the sequence level was observed, except for a region between *At2G30000* and *At2G30020.* The homologue of *At2G30010* was lost from the A3/C3 homoeologous region of *B. napus* and *B. rapa*, and a part of the ‘R’ block that contained the *FLC* homologue and a small part of the ‘U’ block that contained a homologue of *CBF1* (*C-REPEAT/DRE BINDING FACTOR 1*, a cold-response gene) were inserted into this region. BLASTN searches against coding sequences of *A. thaliana* revealed no further evidence for homologous fragments of *FLC* and *CBF1* in those regions (Table S4). This interruption of microsynteny indicates that a multiple rearrangement occurred after the whole-genome triplication of the *Brassica* genomes. We further analyzed a *B. rapa* BAC (KBrH038M21) that contained *BrFLC.A3* from 24.3–24.4 Mb of chromosome A3 [35]. This BAC contained many repetitive sequences, including a few transposon-like sequences (Figure 6B), which suggests that the complex structural rearrangement involved transposon activation.

The genomic sequences of *B. rapa*, *A. thaliana*, and *A. lyrata* were used to examine the microsynteny structure around the *FRI* locus. Surprisingly, in contrast to *FRI* which is located within the ‘O’ block of *A. thaliana*, all the other *FRI* homologues were found in the ‘W’ blocks either on chromosome 8 of *A. lyrata* (AL8_14.3 Mb), which is thought to be more similar to the ancestral karyotype of Brassicaceae (n = 8), or chromosomes A3 and A4 of *B. rapa* (BrA3_W and BrA4_W), respectively (Figure 6A). *BrFRI.A3* was localized approximately 200 kb away from *BrFLC.A3b* according to the genomic sequence (version 1.1) of *B. rapa* database [36]. However, a fragment between the gene models of *At5G51090* and *At5G51000* showed high similarity (approximately 85–98%) to *AtFRI* but lacked exon 1 was found in the‘W’block of *A. thaliana* (At5_20.7 Mb). Consistently, a previous study on comparative mapping of *FRI* loci between *A. lyrata* and *A. thaliana* showed that *AlFRI* was unexpectedly mapped to *A. lyrata* chromosome AL8 rather than AL6, which contained the homologous ‘O’ block [37]. Moreover, a recent research on comparative analysis of *FRI* among *B. oleracea* (*BoFRI*) and the *Arabidopsis* lineage also showed that the two *BoFRI* homologues are located within the regions collinear to the ‘W’ blocks [38]. The results of previous and present studies suggested that the *FRI* homologues located in the ‘W’ block is the same as the gene inherited from the common ancestor of Brassicaceae, and that the *FRI* copy on the ‘W’ block had probably been translocated to the ‘O’ block during the evolution of *A. thaliana.*

To obtain a deeper insight into the coevolution of *FLC* and *FRI* homologues in *B. napus*, we calculated the correlation between the expression of different pairs of *FLC* and *FRI* homologues during different developmental stages. Since we could only design suitable qRT-PCR primers for *BnFRI.A3* and *BnFRI.X* (termed as *BnaA.FRI.a* and *BnX.FRI.c* by Wang *et al*. [31]), the two *BnFRI* homologues along with the four *BnFLC* homologues were examined for their expression levels by qRT-PCR. A significant correlation was observed within different *BnFLC* and *BnFRI* gene pairs (*BnFRI.A3* and *BnFLC.A3b*, *BnFRI.X* and *BnFLC.A2*, and *BnFRI.X* and *BnFLC.A10*) in leaves of nonvernalized Ningyou7 plants (Table 2). Among these tested gene pairs, the closely linked genes *BnFRI.A3* and *BnFLC.A3b* showed a highly significant correlation (Pearson’s correlation coefficient, *r* = 0.965, *P*<0.001) in relative expression levels from the cotyledon stage to the flowering stage in nonvernalized Ningyou7. We investigated whether the correlation of expression was significant in the winter-type cultivar, Tapidor, in the absence of vernalization. Given that Tapidor has an obligate requirement for cold to enter the reproductive phase, we analyzed the correlation of expression during leaf development (the cotyledon to four-leaf stages). Although all *BnFRI* and *BnFLC* pairs showed positive correlation of expression in Tapidor, none of them had significant correlation (p<0.05).

**Table 2:** Correlation coefficients between *BnFLC* and *BnFRI* loci in the Tapidor and Ningyou7 during different developmental stages.

	*BnFRI.A3*	*BnFRI.X*	*BnFRI.A3*	*BnFRI.X*
	Ningyou7^a^	Ningyou7	Tapidor	Tapidor
*BnFLC.A2*	0.599 (0.1249)^b^	0.945 (0.0004)	0.518 (0.1025)	0.313 (0.3492)
*BnFLC.A3b*	0.965 (0.0001)	0.658 (0.0760)	0.370 (0.2634)	0.453 (0.1613)
*BnFLC.A10*	0.544 (0.1702)	0.883 (0.0036)	0.347 (0.2961)	0.149 (0.6624)

a Pearson’s correlation coefficients were calculated by relative expression values within different *BnFLC* and *BnFRI* gene pairs in cotyledons or leaves which harvested from the cotyledon, the stem elongation and the flowering stages of nonvernalized Ningyou7, or from the cotyledon, the first-, two-, and four-leaf stages of nonvernalized Tapidor plants.

b The value in parentheses represents the *P*-value.

## Discussion

We cloned nine *FLC* homologues from the *B. napus* genome and identified their map positions. We analyzed the promoter sequences of these genes in comparison to their homologues from the diploid progenitors *B. rapa* and *B. oleracea,* and in comparison to the *A. thaliana* gene *FLC*. The *BnFLC* homologues were expressed differentially. One *FLC* homologue (*BnFLC.A3b*) was of particular interest because it colocalized with a major QTL for flowering time. Moreover, it was expressed differentially between the parents of our mapping population and was located in the vicinity of the *FRI* homologue *BnFRI.A3*. The progenitor of *BnFLC.A3b* was duplicated ectopically into the ‘J’ block on chromosome A3 and was part of a gene cluster with two functionally related genes, *BnFRI.A3* and *BnCBF.A3*. This gene cluster is present specifically in the *Brassica* lineages.

### *FLC* Homologues in *B. napus* Contribute Mainly to Variation in Flowering Time

We identified nine *FLC* homologues in *B. napus*. In addition to the non-functional *BnFLC.C3b,* six cDNA sequences were found in the winter cultivar Tapidor and in the semi-winter cultivar Ningyou7, and the remaining two (*BnFLC.C2* and *BnFLC.C3a*) were only isolated from the spring cultivar Westar (Table S2). This suggests that most of the homologues were transcriptionally active in different cultivars. Interestingly, flowering-time QTL have been found to coincide with the map positions of *FLC* homologues in different mapping populations, e.g. *BnFLC.A3b* and *BnFLC.A10* (TN-DH population) and *BnFLC.A3a* and *BnFLC.C2* (Skipton/Ag-Spectrum DH population), which clearly suggests a role of these homologues in controlling flowering time in *B. napus.* However, direct links between the expression of *FLC* homologues and each of the flowering-time QTL need to be investigated further.

Four *BnFLC* homologues tested in the present study were expressed ubiquitously before vernalization and were repressed significantly after vernalization, which suggested that they act in a similar manner to the *FLC* gene in *Arabidopsis* [4], [39], [40]. However, the expression patterns of different *BnFLC* copies were quite different. *BnFLC* transcripts were detected in buds and flowers of vernalized Tapidor plants, especially for *BnFLC.A3b* (Figure 4); similar observations were reported for transgenic *Arabidopsis* plants that harboured the *pBoFLC4-1::BoFLC4-1::GUS* construct [41]. These results indicated that reactivation of *Brassica FLC* genes during gametogenesis was similar to that of *FLC* in *Arabidopsis*. In *Arabidopsis*, *FLC* activity is reset in the male reproductive tissue and repressed in maturing pollen in vernalized flower buds [39], although the precise biological roles of *FLC* in these tissues remain unknown. In addition, the expression level of *BnFLC* homologues in leaves of nonvernalized Ningyou7 plants at the stage of inflorescence emergence was as high as during the seedling stage, which suggested that in the semi-winter cultivar Ningyou7, other genes might overcome the repression by *BnFLC* to activate the downstream floral integrators. The high level of expression of *BnFLC* genes even after flowering indicates that *Brassica FLC* homologues might have multiple functions in the regulation of plant development, as reported in *Arabidopsis* [42]. Additionally, *BnFLC.A10* showed more decrease of fold change in Ningyou7 as compared to Tapidor at the early stage of cold-treatment, which is consistent with functional differences of *BnFLC.A10* alleles between Tapidor and Ningyou7, which are located in the major flowering-time QTL (TN-DH population). Their expression decreased rapidly in Ningyou7 leaves during vernalization (Hou *et al*., unpublished data), suggesting that some *BnFLC* homologues respond in a more sensitive way to vernalization in the early flowering cultivar (Ningyou7).

Although *FLC* homologues were highly conserved among *B. napus* and its diploid progenitors *B. rapa* and *B. oleracea,* the promoter sequences among *Brassica FLC* genes that belonged to different clades were quite variable and also diverged substantially from that of *AtFLC.* Variations in *cis*-regulatory sequences that affect their function might contribute to phenotypic diversity among different species [43]. In the present study, only five *cis*-blocks were identified in the promoter of the *BnFLC* homologues and *AtFLC*, which suggested that these segments might require for common regulation of *FLC* homologues. Furthermore, we identified a more highly conserved 30-bp region within *cis*-block 4, which together with the presence of the newly discovered *cis*-block 5 in most *FLC* homologues indicated that important *cis*-regulatory element(s) might be present in these regions. The *BnFLC* sequences were highly conserved among the cultivars Columbus [28], Tapidor, and Ningyou7 at the coding sequence level; thus, the divergent promoter sequences might contribute to the differential regulation of *Brassica FLC* homologues and explain their functional divergence. Consequently, a more sophisticated network of interactions among *FLC* homologues and their targets might have evolved in *B. napus* as compared with *Arabidopsis*.

### Functionally Related Flowering-time Genes are Tightly Linked in *Brassica* Species

Gene homologues usually maintain collinearity among closely related plant species, for example the extensive sequence conservation identified within the Brassicaceae [19], [20]. In the present study, *BnFLC.A3b*/*BnFLC.C3b* was located at one end of the ‘J’ block on chromosome 3, in contrast to *AtFLC* and other *BnFLCs*, which were located within the ‘R’ block. A microsynteny analysis of *FLC*-related segments in *Brassica* and *Arabidopsis* lineages suggested that the ancestral homologue of *BnFLC.A3b* in *Brassica* was inserted into the ‘J’ block on chromosome 3. In addition, a neighbour-joining analysis revealed that *BnFLC.A3b*/*BnFLC.C3b* and *BnFLC.A3a*/*BnFLC.C3a* belong to the same clade (Figure 1). These results suggest that *BnFLC.A3b*/*BnFLC.C3b* might be derived from a common ancestor of *BnFLC.A3a*/*BnFLC.C3a*, and was duplicated ectopically at the present loci during the period between the genome triplication of *Brassica* and the divergence of *B. rapa* and *B. oleracea*. Similarly, a homologue of *CBF1* was also translocated from the ‘U’ block; *CBF1* is a cold- and dehydration-responsive gene [44], is regulated negatively by *SOC1*, and probably interacts with *FLC* [42], [45]. The present study and recent comparative analysis of *FRI* homologous regions between *B. oleracea* and the *Arabidopsis* lineage [38] revealed that the ancestral *FRI* was located originally in the ‘W’ block. Chromosomal rearrangements during the evolution of *Brassica* genome, led to separated blocks from different chromosomes of the ancestral karyotype which are recombined among each other and which are not shared with *A. thaliana* genome [46], e.g. the blocks ‘W’ and ‘J’ on the homologous chromosome A3/C3 from the *Brassica* genome. Thus, the three unlinked functionally related homologues of *FLC*, *FRI*, and *CBF1* were typically clustered within a short distance in the *Brassica* A genome, and the clustering event involved at least two translocations and a reorganization of the ‘J’ and ‘W’ blocks on *Brassica* chromosome A3 (Figure 6).

Increasing evidence demonstrates that functionally related genes tend to cluster together in several eukaryotes and this clustering might be maintained by selection pressure [47], [48]. Recently, several gene clusters involved in secondary metabolic pathways that presumably confer a selective advantage in nature have been discovered in plants [49]–[51]. The clustering of *FLC–FRI*–*CBF1* in *Brassica* might be subjected to selection to adjust the flowering time in biennial plants. Although we identified a major flowering-time QTL adjacent to the *FLC–FRI–CBF1* region (in the TN-DH population) and detected higher expression of the *BnFLC.A3b* allele from the later-flowering parent (Tapidor), neither sequence variation in the regulatory regions, nor different DNA methylation level from the core promoter to the 5′ end of intron 1 were observed between the *BnFLC.A3b* alleles of Tapidor and Ningyou7. In *Arabidopsis*, *FRI* upregulates expression and promotes efficient splicing of *FLC* via a cotranscriptional mechanism [10]. Although the expression levels of each *BnFRI* homologue were similar between Tapidor and Ningyou7, nonsynonymous nucleotide substitutions, which are located within the N-terminal putative coiled-coil domain of the *BnFRI.A3* allele in Ningyou7, probably contribute to functional differences between Tapidor and Ningyou7 [31]. Therefore, the splicing variants of *BnFLC.A3b* probably arise because of the presence of the weaker functional allele of *BnFRI.A3* in Ningyou7, which is less capable of upregulating *FLC* and promoting accurate splicing. Although the precise role of *BnFRI.A3* in altering the expression of *BnFLC.A3b* remains unknown, adjacent genes that are localized within the same chromatin domain can be regulated coordinately [52]. A tendency for coexpression of *BnFRI.A3* and *BnFLC.A3b* at different developmental stages in nonvernalized Ningyou7 plants further suggested that the adjacent *BnFRI.A3* and *BnFLC.A3b* genes probably interact, although it was not obvious in Tapidor. This result probably reflected the fact that the samples of Tapidor used for analysis could only be harvested at stages of leaf development, but alternatively the winter-type cultivar might harbour more strongly functional genes that affect the expression of *BnFRI* and *BnFLC* homologues. Thus, regardless of whether the *FLC–FRI*–*CBF1* cluster in the *Brassica* lineage was assembled randomly or driven by evolution, this cold-responsive gene cluster in *B. napus* might be maintained by selection pressure and thus enable adaptation to the extremely diverse environments under which *Brassica* crops are cultivated worldwide.

## Materials and Methods

### Plant Materials

The *B. napus* winter-type cultivar Tapidor which has an obligate vernalization requirement, semi-winter-type cultivar Ningyou7 which has a weak vernalization requirement, and spring-type cultivar Westar were used for cloning and/or expression analysis of *BnFLC* genes. The TN-DH population, which comprised 202 doubled-haploid lines, was derived from the F_1_ cross between Tapidor and Ningyou7 [53]. The Skipton/Ag-Spectrum DH population which comprised 186 doubled-haploid lines was developed from the cross between spring-type Australian cultivars, Skipton and Ag-Spectrum at the Wagga Wagga Agricultural Institute, Australia [54]. Both DH populations were used for genetic mapping and detection of QTL for flowering time. Plants were grown in the field that are either belong to Huazhong Agricultural University or to NSW Department of Primary Industries and only used for phenotyping or DNA/RNA extraction.

### Cloning and Sequence Analysis of *BnFLC* Homologues

To isolate genomic sequences of the *BnFLC* homologues, a BAC library (JBnB) constructed from the gDNA of Tapidor [20] was used. A fragment of *BnFLC.A3a* (exon 2 to exon 6) was used as a probe to screen the JBnB BAC library by Southern blot hybridization. Different *BnFLC* homologues were identified by sequencing the PCR products that were amplified from the BAC clones. The PCR primers used are listed in Table S1.

To isolate the cDNA sequences of the *BnFLC* homologues, 2 µg of total RNA that had been extracted from four-week-old leaves of Tapidor, Ningyou7, or Westar was used to synthesize first-strand cDNA with the First Strand cDNA Synthesis Kit (Fermentas, Glen Burnie, MD, USA). Gene-specific primer pairs were designed on the basis of an alignment of the *Brassica FLC* cDNA sequences deposited in GenBank (AY036888–AY036892, DQ866874–DQ866876, AY115678, AM231517, AM231519, AY306122, and AY306123). The existing nomenclature for *Brassica FLC* genes is unsuitable to distinguish different *FLC* homologues from the two subgenomes (AA and CC); therefore, we used a new nomenclature to describe the *Brassica FLC* genes on the basis of their map position on different linkage groups. For example, *BnFLC.A3a* denotes the *FLC* gene of *Brassica napus* (*Bn*) that maps to linkage group (chromosome) 3 of the A genome; an additional suffix (a or b) is used if the same linkage group contains more than one *FLC* homologue.

The alternatively spliced transcript of *BnFLC.C3a* was isolated from leaves of *B. napus* cv. Westar with GeneRacer kit (Invitrogen Corporation, Carlsbad, CA, USA) using degenerate 3′ RACE primers (Table S1). KOD-Plus DNA polymerase (Toyobo Co., Osaka, Japan) was used for PCR amplification, and additional deoxyadenosine nucleotides were introduced at the 3′ end of amplicons using Taq DNA polymerase (Fermentas). Amplicons were ligated to the pGEM-T vector (Promega Corporation, Madison, WI, USA) and transformed into *Escherichia coli* strain DH5α. Positive clones were selected and sequenced by the Nanjing GenScript Co., Jiangsu, China. Sequence data from this article have been deposited in the GenBank database with the following accession numbers: JQ255381 to JQ255389 for genomic sequences of *BnFLC* homologues, and JQ255390 to JQ255397 for the corresponding mRNA sequences.

Potential conserved *cis*-blocks and *cis*-elements of *FLC* homologues were identified by Dna Block Aligner (http://www.ebi.ac.uk/Tools/Wise2/dbaform.html, validated on 30^th^ July, 2012), after removal of the simple repetitive sequences by Repeatmasker (http://www.repeatmasker.org), and potential *cis*-elements were predicted by PlantCARE (http://bioinformatics.psb.ugent.be/webtools/plantcare/html/, validated on 30^th^ July, 2012). The GenBank accession numbers of the *BrFLC* and *BoFLC* genes that were used for analysis are: *BrFLC.A10* (*BrFLC1*), AC155344; *BrFLC.A2* (*BrFLC2*), AC155341; *BrFLC.A3a* (*BrFLC3*), AC155342; *BrFLC.A3b* (*BrFLC5*), AC232559; *BoFLC.C9* (*BoFLC1*), AM231517; *BoFLC.C2* (*BoFLC4*), AY306124; *BoFLC.C3a* (*BoFLC3*), AM231518; *BoFLC.C3b* (*BoFLC5*), AM231519.

### Genetic Mapping of *BnFLC* Homologues and QTL Detection

On the basis of allelic polymorphisms between Tapidor and Ningyou7, gene-specific PCR primers for *BnFLC.A3a* and *BnFLC.A3b* were designed, and a single-strand conformation polymorphism marker for *BnFLC.C2* was developed on the basis of the sequence of the *BrFLC2-*containing BAC (KbrH004D11, Table S1). The genotypic data for *BnFLC.A3a*, *BnFLC.A3b*, and *BnFLC.C2*, and those reported previously for *BnFLC.A10*, *BnFLC.C3a*, and *BnFLC.C3b* [29], were incorporated into the map data for the TN-DH population [55] using JoinMap 3.0 software (http://www.kyazma.nl/index.php/mc.JoinMap). Common markers between a consensus genetic linkage map [56] and the TN-DH genetic map were used as anchors to integrate the RFLP markers of FLC2aH (*BnFLC.A2*) and FLC1aH (*BnFLC.C9*) into the TN-DH genetic map and for alignment with the physical position of *FLC* in *Arabidopsis*. The integrated map, based upon 786 molecular markers, was used to locate QTL for flowering time by the composite interval method with WinQTL Cartographer 2.5 software [57], [58] using flowering-time data collected previously from 12 winter- (with cold treatment) and two spring-crop (without cold treatment) environments [29], [59].

For the Skipton/Ag-Spectrum DH population, the published molecular linkage map comprising 674 markers was utilized to detect flowering time QTL associated with *BnFLC* homologues. Flowering time was scored under glasshouse and field conditions as described previously [60], [61].

### Gene Expression Analysis and Quantitative Real-time PCR (qPCR)

To analyze the spatial expression patterns in response to vernalization of different *BnFLC* homologues, Tapidor plants were grown in a greenhouse at approximately 25°C under long-day conditions (14 h light/10 h dark). For vernalization treatment, four-week-old plants were incubated for three weeks in a climate chamber maintained at approximately 5°C under the same long-day conditions. Cotyledons were harvested 5 days after sowing; leaves, stems, and roots from nonvernalized Tapidor plants were harvested at the four-leaf stage; leaves, stems, floral buds, and flowers from vernalized Tapidor plants were harvested at the flowering stage under the field conditions. To analyze changes in the expression of *BnFLC* homologues among different developmental stages and the correlation in expression between *BnFLC* and *BnFRI* genes, we used the same set of cDNA samples from leaves of Ningyou7 plants that was used by Wang *et al.* [31]. For the expression correlation analysis in Tapidor, cotyledons and leaves at the first-, two-, and four-leaf stages from nonvernalized Tapidor plants were used.

Gene-specific primers were designed on the basis of the cDNA sequences of the *BnFLC* homologues with Primer Premier 5.0 (Premier Biosoft International, Palo Alto, CA, USA; Table S1), and tested by melting curve analysis and sequencing. Total RNA was extracted from different samples with the Quick Extract™ RNA isolation kit (BioTeke Corporation, China). qRT-PCR was performed on a CFX96 Real-Time System (Bio-Rad Laboratories, Hercules, CA, USA). Reactions were performed in a final volume of 20 µL with SYBR Green qPCR SuperMix-UDG with ROX (Invitrogen Corporation, Carlsbad, CA, USA) as described previously [31]. Three to four technical replications and three biological replicates were performed for each sample. For data analysis, the relative standard curve method was used [62]. A standard curve was prepared on each PCR plate for each primer pair using five 10-fold serial dilutions of plasmid DNA to determine the amplification efficiency. Quantification cycle (C_q_) values were calculated and the relative expression values of target genes were normalized with the reference gene *β-Actin* [31] using the CFX Manager 1.5 software (Bio-Rad). Subsequently, the data was exported to SAS 8.0 (SAS Institute, 1999) for statistical analysis.

### Detection of DNA Methylation

Genomic DNA was extracted from leaves of nonvernalized plants. Approximately 1 µg of DNA was treated with the EpiTect Bisulfite Kit (Qiagen, Germany) in accordance with the manufacturer’s instructions. Gene-specific primers (Table S1) were designed with MethPrimer [63]. Stable unmethylated endogenous gene controls [64] were used to estimate whether the bisulfite treatment was completed. At least 10 clones were sequenced for each PCR product.

### Comparative Genome Analysis

Two BACs (JBnB142O14 and JBnB090B17), which contained *BnFLC.A3b* and *BnFLC.C3b*, respectively, were sequenced in different pools that contained several *B. napus* and *B. oleracea* BACs. Each pool was sequenced with a Genome Sequencer FLX system (Roche, Germany). The obtained raw sequence data were assembled using the GS De Novo Assembler (Newbler) 2.5 software (-v pBACsacB1 -s ecoli). Assembled contigs were submitted to a BLAST search against KBrH038M21 (which contains *BrFLC5*) and highly homologous contigs were selected for further analysis in this context.

The genomic sequences of *A. thaliana*, *A. lyrata*, and *B. rapa* were downloaded from The *Arabidopsis* Information Resource (TAIR; http://www.arabidopsis.org/), the Department of Energy Joint Genome Institute (JGI; *A. lyrata* version 1.0 http://genome.jgi-psf.org/Araly1/Araly1.home.html), and the *Brassica* Database (BRAD; http://brassicadb.org/brad/index.php). The sequences of the four BACs that contained *BrFLC* (KBrH080A08, KBrH004D11, KBrH117M18, and KBrH052O08) were used. The sequences of the analyzed segments were reannotated in detail using Genscan (http://genes.mit.edu/GENSCAN.html) and submitted to a BLAST search against TAIR to identify homologous genes in *Arabidopsis*. Predicted proteins that were less than 100 amino acids in length and had no hits in the target database were excluded from the final annotation. Homologous fragments that were similar to coding sequences in *Arabidopsis* but were not incorporated into gene models were identified using the BLASTN tool [65]. Repetitive sequences were identified by lower-case letters and were submitted to a search against the Plant Repeat Databases (http://plantrepeats.plantbiology.msu.edu/index.html) and Repeatmasker (http://www.repeatmasker.org) for comparison with known repeat sequences in plants.

### Phylogenetic Analysis

A multiple alignment of relative sequences was generated with CLUSTALW [66]. Ambiguously aligned regions were modified manually to minimize the number of inserted gaps. Phylogenetic trees were constructed with MEGA 5.0 software [67] using the neighbour-joining method with the ‘partial deletion’ option for analysis of gaps. A bootstrap analysis with 2000 replicates was used to evaluate the robustness of the tree topology.

## Supporting Information

Figure S1 (A) Alignment of cDNA sequences of *BnFLC* homologues and their relative orthologues in *B. rapa* and *B. oleracea*. The accession numbers of the *B. rapa* and *B. oleracea* sequences are shown in parentheses. Additional nucleotides in exon 2 and exon 7 of *BnFLC.C3b* and *BoFLC.C3b* (*BoFLC5*; GenBank accession No. AM231519) are highlighted with arrows. (B) Gene structure of *BnFLC.A3b*. The positions of exons and untranslated regions (UTRs) are represented by boxes.
(TIF)

Figure S2 (A) cDNA and (B) amino acid sequence alignment of alternatively spliced variants and their predicted polypeptides of *BnFLC.A3b* in Tapidor and Ningyou7. *BnFLC.A3b_T, N* represents a correctly spliced transcript in Tapidor and Ningyou7, *BnFLC.A3b_N-1* and *BnFLC.A3b_N-2* represent the two kinds of alternatively spliced transcripts in Ningyou7 which are missing exon 3 partly or completely.
(TIF)

Figure S3 **Genome blocks and the location of** ***FRI*** **,** ***FLC*** **, and** ***CBF*** **homologues in** ***Arabidopsis thaliana*** **(At) and** ***Arabidopsis lyrata*** **(Al).** Arrows indicate the opposite orientation of the blocks.
(TIF)

Table S1 **Primers used for cloning, mapping, expression, and DNA methylation analysis.**
(XLS)

Table S2 **Information for isolated cDNA sequences of** ***BnFLC*** **homologues.**
(XLS)

Table S3 **Comparison of methylated sites in the predicted CG islands of** ***BnFLC.A3b*** **between the Tapidor and Ningyou7 alleles.**
(XLS)

Table S4 **Summary of BLASTN analysis to detect similar coding sequences in the** ***Arabidopsis*** **genome.**
(XLS)
