# Supplementary figures and images for: Comparative Analysis of FLC Homologues in Brassicaceae Provides Insight into Their Role in the Evolution of Oilseed Rape
Source: PLoS One. 2012 Sep 27;7(9):e45751. doi: 10.1371/journal.pone.0045751 (PMC3459951; doi:10.1371/journal.pone.0045751)

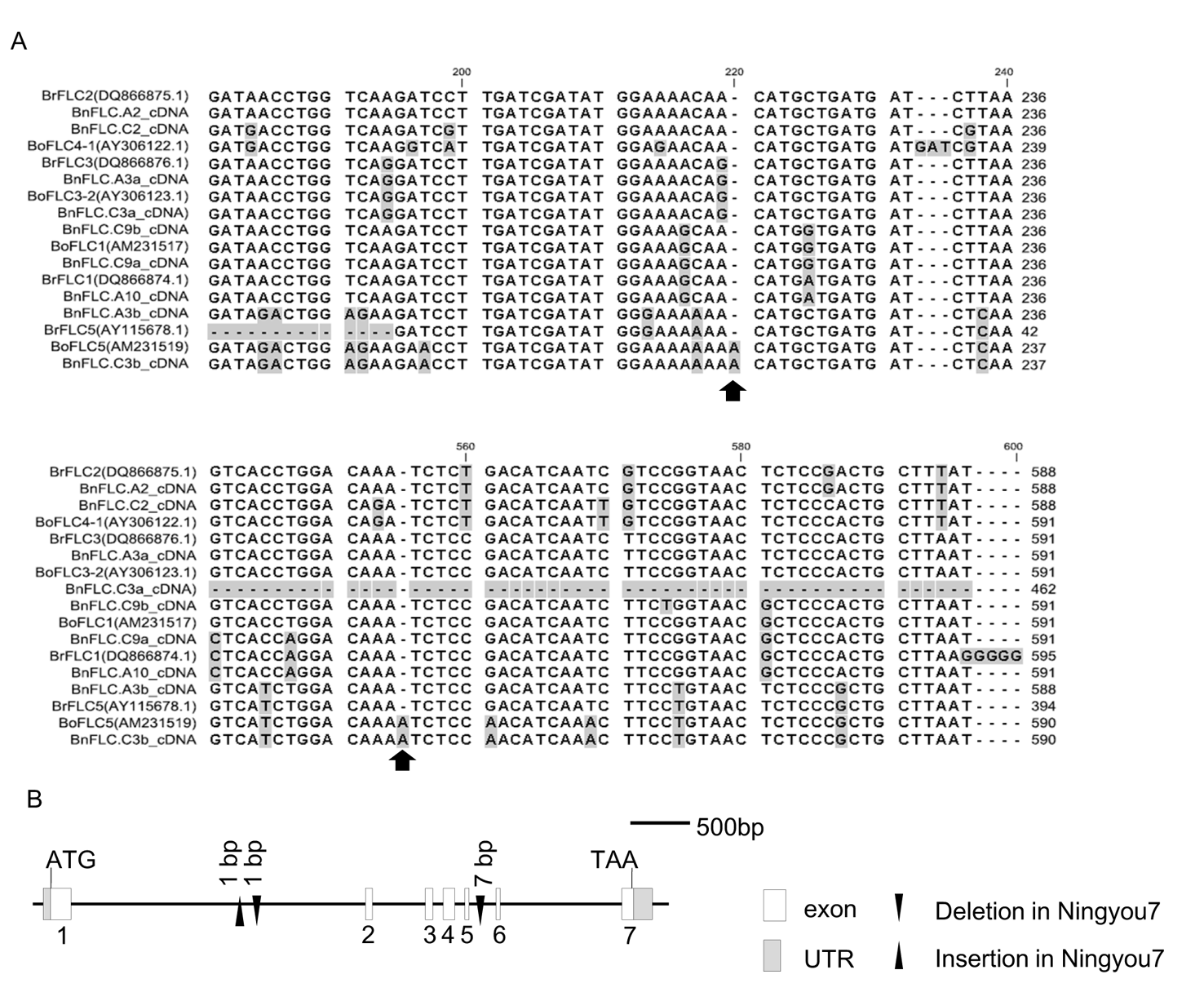

Supplement: Figure S1 — (A) Alignment of cDNA sequences of BnFLC homologues and their relative orthologues in B. rapa and B. oleracea. The accession numbers of the B. rapa and B. oleracea sequences are shown in parentheses. Additional nucleotides in exon 2 and exon 7 of BnFLC.C3b and BoFLC.C3b (BoFLC5; GenBank accession No. AM231519) are highlighted with arrows. (B) Gene structure of BnFLC.A3b. The positions of exons and untranslated regions (UTRs) are represented by boxes. (TIF) [file pone.0045751.s001.tif]

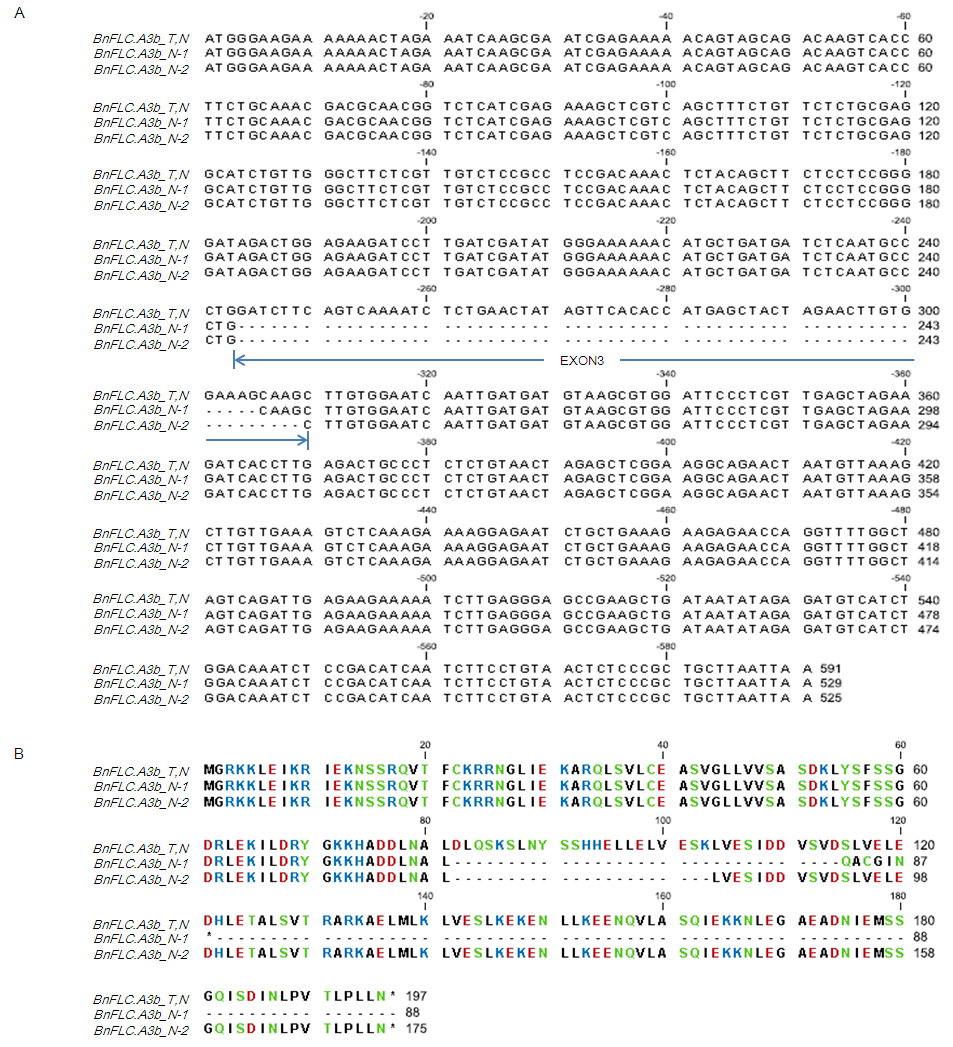

Supplement: Figure S2 — (A) cDNA and (B) amino acid sequence alignment of alternatively spliced variants and their predicted polypeptides of BnFLC.A3b in Tapidor and Ningyou7. BnFLC.A3b_T, N represents a correctly spliced transcript in Tapidor and Ningyou7, BnFLC.A3b_N-1 and BnFLC.A3b_N-2 represent the two kinds of alternatively spliced transcripts in Ningyou7 which are missing exon 3 partly or completely. (TIF) [file pone.0045751.s002.tif]

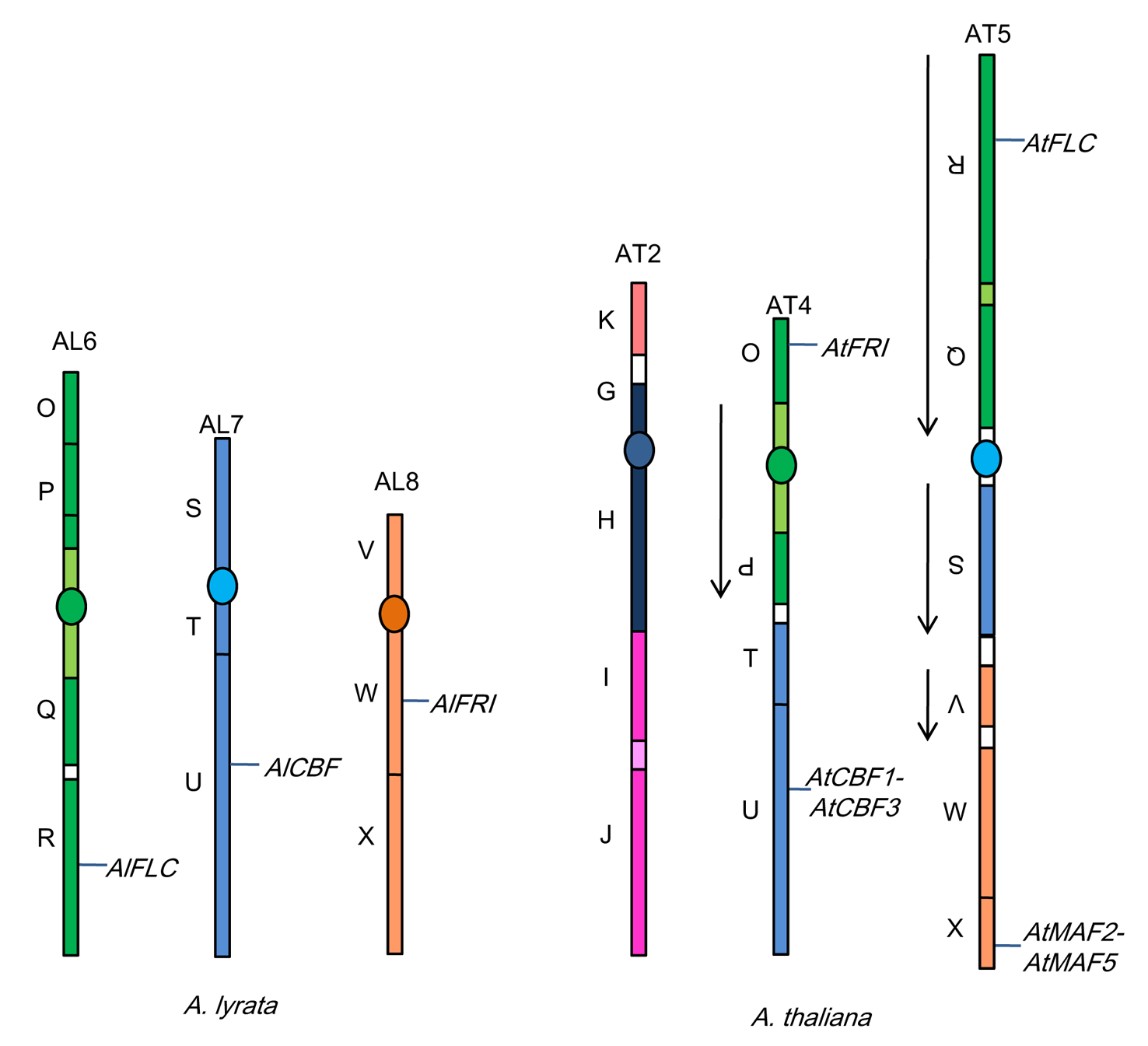

Supplement: Figure S3 — Genome blocks and the location of FRI , FLC , and CBF homologues in Arabidopsis thaliana (At) and Arabidopsis lyrata (Al). Arrows indicate the opposite orientation of the blocks. (TIF) [file pone.0045751.s003.tif]
